# Notes on the Practice in the Hospitals of Paris

**Published:** 1858-07

**Authors:** George Suckley

**Affiliations:** Late Assistant-Surgeon, United States Army


### SELECTIONS.

*Notes on the Practice in the Hospitals of Paris. The Ecraseur," "Electric cautery," Maisonneuve's method of dividing strictures, etc., etc.* By George Suckley. M. D., late Assistant-Surgeon, United States Army.

In watching the practice at the various Parisian Hospitals, we find many methods in vogue at one or the other, which are either entirely new or else but partially known to members of the Medical Profession in the United States. Some of these plans of treatment appeared confined, for the most part, to their originators, or to persons who, having adopted them, ride their "hobbies" on all occasions. They would, for example, *if possible,* amputate with a "drainage tube," tie an artery with an "écraseur," or trephine with the actual cautery, after all of which applications, most plausible reasons would be assigned by the enthusiastic operator showing, to his own satisfaction at least, the eminent superiority of his plan of treatment over all others.

Some of the prevailing "hobbies," however, have their advantages in special cases, and are, therefore, entitled to our careful attention, and, although descriptions of several have been more or less perfectly given in the different American Medical Journals, it may not be amiss to describe the manner in which they are at present applied in Paris, even at the risk of repetition.

*The "Ecraseur."*—The use of the "écraseur" is confined to two hospitals, La Pitié and the Lariboisière. In the latter Chassaignac every Monday morning expatiates upon the merits of *"l' ècrasement linéaire,"* accompanying his oratory, as far as possible, with practical manifestations of its powers. In his hands the instrument certainly works admirably in many cases, especially in operations about the anus, tongue, cervix uteri, or vagina, or in almost any other situation when there is danger of excessive hemorrhage, as well as in cases where the loss of even a small quantity of blood cannot well be borne.

The surgeon above named gives the following summary of the benefits accruing from the use of this instrument, as compared with the knife:

1st. *The almost total absence of hemorrhage.*

2nd. *The rarity with which the employment of this instrument is followed by profuse suppuration, erysipelas, or* PHLEBITIS AND ITS CONSEQUENCES.

It is almost needless to say that union by first intention is almost entirely done away with when this instrument is used. As offsets to this, are the facts that union by first intention is rarely got in the Paris hospitals after the knife, and even if obtained, it is of course of but little account when compared to the excessive frequency of "purulent absorption."

Among at least a dozen operations that I have seen performed with this instrument, there was one which pleased me very much. This was the removal of a hemorrhoidal tumor of about the size of a pigeon's egg, situated just within the verge of the anus. The tumor having been pulled down with a peculiar "clawed" instrument, the chain of the "écraseur" was passed around its base, and drawn moderately tight by hand. The instrument was then "worked" in the usual manner by raising and depressing its pump-like handle, each elevation or depression being accompanied by a sharp "click," at the same time drawing in about a twelfth of an inch of the chain. The instrument was worked very slowly, eight minutes elapsing between the commencement of the operation and the complete separation of the tumor. The amount of hemorrhage during, and at the end of the operation, would sarcely fill three teaspoons. It took twenty-four elevations and depressions of the lever of the instrument to execute the operation. The patient, being under the full influence of chloroform, was operated upon while reclining on his side.*

Outside of the Lariboisière the "écraseur" does not appear to be a favorite in the Paris hospitals, with the single exception of La Pitié, where Maisonneuve uses it not unfrequently, and carries its application with his usual ardor, or recklessness, to an extreme. He has attempted

* The "ecraseur" although now well known to many in the United States, is, perhaps, a sort of unintelligible article to some. For the benefit of such, the following rough explanation may be kindly received: Suppose you wish to divide a sphincter ani by this method when operating for fistula in ano—Take an ordinary chain-saw with very blunt teeth, pass one end up the fistula, and bring it out through the anus. This being done, draw the two ends through a strong metallic double canula or tube. Then by means of a small windlass at the further end of the tube, tighten the chain until it has, in the course of ten minutes, cut through the sphincter, and is entirely free. Thus you have planned, put together, and operated with an extemporaneous "ecraseur which contains in principle all the elements of one of "Charriere's best," but which lacks, of course, finish, strength, and the improved power that an instrument made by a professional instrument-maker posseses.

to amputate with it several times, but the details or results of these operations are far from being perfectly satisfactry; although the number of such amputations is yet to small too allow the formation of a practical conclusion of the general merits of the process. In the last case it was used for the amputation of an arm at about its middle, but the screw of the *bones breaker* (I know not what else to call it) caused the bone to snap at a point some distance above the insertion of the deltoid, and, in endeavoring to twist the loosened extremity clear from the insertion of the muscle, the wound was forced open, and the brachial artery commencing to bleed freely, had to be tied. The unfortunate patient died after several weeks, but it was thought that his death was more properly owing to an independent disease of the lungs, and not a direct sequence of the operation.

(Since writing the foregoing, another amputation with the "écraseur" has taken place in time for me to insert its details before mailing this paper):

*Amputation of the forearm with the "écraseur."*—A girl of 17, employed in a manufactory of percussion caps, was, on the 1st of February, severely injured by the explosion of a large number of these articles. Among other injuries more or less severe, the right hand was so shattered, that on the day following the accident Maisonneuve decided upon amputating the lower end of the forearm. The girl having been rendered unconscious by chloroform, the operation commenced. The first desideratum was to break the bones at a point about two inches above the wrist, which was accomplished by means of a screw working in a complicated set of machinery that I cannot attempt to describe in words.—The next thing was to apply the "écraseur" sufficiently below the point where the bones were broken, to give sufficient covering to the stump.—After this latter instrument had its chain considerably tightened upon the portion that it surrounded, an incison was made around the forearm at a point just above the wrist, and the lower fragments twisted and pulled through it. The chain of the "écraseur" was then tightened, and the operation completed. No tourniquet was used, no vessels tied, and not a teaspoonful of blood lost.

As the patient was in a better state of anæsthesia than is common to find in Paris, she only shrieked moderately during the middle of the operation. The time consumed during the amputations was about 15 minutes; that from the commencement of the working of the 'écraseur" *six minutes*. The advantages expected to result from the operation are as follows; 1st. Less hemorrhage, as we have seen. 2nd. Less danger of

*phlebitis*—a very important consideration to surgeons of crowded hospitals.

The ultimate result of the case will, of course, be the best proof for or against the sagacity of the measure.

The operation, as a *spectacle*, seems very barbarous. If the patient is well under the influence of the anæsthetic, the semblance of brutality is greater than the reality; and if the operation is more successful than the others the end ought to justify the means. It is well known that phlebitis, and the so-called "purulent absorption," are the great evils with which the Parisian surgeons have to contend. Of late years nearly all the larger amputations have proved fatal from these causes. Ma sonneuve, in reflecting upon the wretched condition of affairs, came to the conclusion that no means could be more disastrous in result than the ordinary employment of the knife in these operations. He therefore considered it his duty to cast about for some other means of obtaining a successful process.

After the "écraseur" had been employed for some time in removal of tumors, for dividing fistulæ, etc., etc., and it had become evident that the amount of cases of purulent absorption was much reduced in proportion, he imagined that could he apply the same method to the removal of the limbs, that he would be justified in expecting more satisfactory result. How to sever the bones became the next consideration. Viewing the comparative infrequency of purulent absorption as a sequence to a compound fracture, he concluded to avail himself of the idea thus thrown out, and by following his object pertinaciously, he has after many experiments, and the trial of eight or nine different kinds of apparatus, at length brought this method of operation to a moderately satisfactory position.

It seems to me, however, that the common employment of this method of amputation will scarcely answer in our democratic country, and even in France, had it not been for the discovery of anæsthetics, the process would never have come within the scope of the wildest flight of a surgical imagination.

There are two principal models of the "écraseur" in use. That of Chassaignac is worked by means of the pump-like handle already spoken of, each elevation and depression of which draws in a certain fixed amount of the chain.

The other is the improved machine of Maisonneuve, by which the power is applied by a screw. This latter instrument has the following advantages:

1st, it has more power; 2d, it can be worked more slowly and not by jerks; 3d, it is not fatiguing to the operator; 4th, it is more simple in its construction, and therefore more readily cleaned or repaired; 5th, it is cheaper.

Maisonneuve's "écraseur" for *amputating* is of extra size, and much greater strength. The ordinary chain is replaced in this instrument by a sort of rope made of tough, strong iron or steel wire.

The following are the prices of these instruments at Charriére's

| | | | | |
|---|---|---|---|---|
| Chassaignac's "écraseur" | | | 45 | francs. |
| Maisonneuve's | " | ordinary size | 30 | " |
| " | " | " with attachment admitting the use of chains two sizes, two chains being furnished. | 45 | " |
| " | " | for amputations—large size | 80 | " |
| " | " | " with the "bone-breaker." as at present used. | 150 | " |
| Extra chains for the common-sized instruments, each | | | 5 | " |

*Chassaignac's "drainage tubes."*—Chassaignac is very fond of introducing small tubes of india-rubber, perforated on their sides, by which he gradually draws off the pus of an abscess. He applies them more particularly to the treatment of the chronic or sub-acute abscesses so commonly met with among the poor of large cities. The modus operandi of applying the "drainage tubes" is to transfix the abscess with a long trocar inclosed in its canula. This being done, the trocar is withdrawn, and the flexible tube passed through the canula in its place. The canular is then removed, leaving the abscess now only transfixed by the drainage tube, the ends of which are then tied together, and the tube thus left until it is thought advisable to remove it permanently, or for the purpose of substituting another. The drainage tubes vary in calibre from ⅛ to nearly ¼ of an inch, their internal diameter varying from that of a knitting needle to that of a No. 3 Hutchinson's bougie. A common elastic catheter of small size, with holes cut along its sides, will answer very well as an extemporaneous drainage tube. In effect, these drainage tubes are simply *hollow india-rubber setons.* They draw off the pus very well *at first,* but after a short time the most of this fluid passes *along the side of the* tube, as is the case with the ordinary *seton.*

An interne in Chassaignac's service says, that frequently after removing a tube and slitting it open, the interior is found *dry!* But this is not apparent to the vision of the man whose hobby it is.

I have not seen "drainage tubes employed in any other hospital than the Lariboisiére. Chassaignac says that their use in the treatment of hydrocele is equal, if not superior, in practical advantages, to the ordinary method of iodine injection.

*The surgical uses of the perchloride of iron.*—In the treatment of *varices* this article is now extensively used, and it has also been employed for the rapid coagulation of the contents of aneurismal sacs. The strength of the solution used, is that recognized in French Pharmacy as of the "thirtieth degree." (This is by weight, 16 parts of the salt to 84 of water.) *Weaker than this, the coagulating effect is lost*, and if used much stronger, it acts as a *dangerous caustic* The solution should be made just before using, because if kept four or five days, it decomposes.

I witnessed an operation for the *radical cure of varices* by this method, at La Pitié. The veins on the lower limbs were markedly dilated and tortuous, certain of the dilatations along the course of the long saphena being as large as an ordinary thimble. In these larger dilatations, the quantity injected was five drops, thrown in as usual by the pecular syringe used, by which every turn of a screw injects exactly half a drop, thus easily regulating the amount employed. In places where the varices were smaller, a less amount was used. Coagulation was almost instantaneous, and the ultimate result of the case very satisfactory.

In operating for varices by this method, it is necessary to compress the veins by a bandage on the cardiac side of the point of operation, as in ordinary cases of venesection. This has a doubly beneficial effect; first, by guarding against the risk of throwing such a dangerous solution into the general circulation; secondly, by causing the veins *to dilate well*, the clots are *larger*, and thus more thoroughly obstruct the circulation through the vein.

I saw the property that the perchloride of iron has of causing instantaneous coagulation put to a novel but very useful purpose by Maisonneuve. This surgeon, during the course of the removal of a cancerous tumor in the submaxillary region, finding that a continuous oozing from the capillaries and smaller blood vessels, was not only obstructing his view, but also weakening, the patient, who was not in a condition to stand the hemorrhage, called for a solution of the article under consideration with which he sponged the wound, with the effect of instantly controlling the hemorrhage.

*The treatment of strictures by dividing from within—Maisonneuve's present method*—This winter, during my observation of the practice in Maisonneuve's "service," at La Pitié, there have been two cases of very

"tight" strictures of the membranous urethra, treated by *internal section.* The *idea,* of course, is by no means *new,* but the peculiarities of the method of operation, employed by Maisonneuve, are well worth attention.

In both the cases which came under my immediate cognizance, the strictures were excessively constricted. The following are the brief details of the plan pursued: the patient lying in the usual position, an elastic capillary bougie was introduced, after some difficulty, through the stricture. This bougie was slightly tapering, having upon its greater end a metallic "ferrule." As soon as the stricture had been fairly passed, a small-sized silver instrument, looking like a nearly straight catheter, was screwed on to the metallic ferrule of the bougie, and then gradually introduced into the urethra, pushing the bougie before it, so that the latter curled itself up in the bladder, after having acted as a pioneer or guide for the silver instrument. The silver instrument having passed through, or *well into,* the stricture, is held steadily until a stylet sliding in a groove is passed down the catheter to the stricture, which it *partially* divides. The cutting instrument has an almondshaped edge, cuts from the *lower side* of the grooved canula and *not* from its *end* as *that* is obstructed by the insertion of the ferrule of the bougie. The stricture being now partially divided, the whole apparatus is then withdrawn, and another flexible bougie, which is generaly *conical,* having the calibre of its main body considerably larger than the bougie first used, is then passed in the same manner as the preceding, and with about the same ease, owing to the already partially divided condition of the stricture. The *large* urethrotome is then screwed up the bougie, as this other was in the first instance, and pushing in the same manner the bougie before it, enters the stricture. The urethrotome being passed sufficiently *through* the constriction, the concealed blade is opened, and the stricture divided completely.—The urethrotome and bougie are then removed, and a large-sized steel sound readily introduced. The whole method strikes the eye as being preferable to Syme's operation of section from *without,* as it does away greatly with the danger of being followed by those distressing sequences, *urinary fistulæ.*

Unfortunately, one of the two cases that I have seen, where this operation was employed, was fatal; the other recovered. First and last, Maisonnenve has thus operated upon a considerable number of cases, but with results far less satisfactory than would be at first supposed. A highly intelligent interne attached to Maisonneuve's "service," informs me that the proportion of deaths is one out of every *four,* thus treated; and that, with the exception of arthritis and delirium, there are no well-marked symptoms before death. The soft parts in the vicinity are neither gangrenous nor unduly inflamed. The same medical gentleman gave it as his opinion, from the autopsies that he has made, that the cause of death is probably the direct absorption of urine into the tissues, acting as a poison. But it is difficult to realize that the small amount of urine thus absorbed would produce such a general poisoning effect, especially when the amout extravasated is not snfficint even to in duce gangrene.—The whole modus operandi of the poisonous agency is wrapped in obscurity, although I suspect that the unfavorable terminations are more or less assisted by the pernicious atmosphere of the hospital wards.

The instrument used by Maisonneuve are manufactured and sold by Charrière at the following prices;

| | | |
|---|---|---|
| Small Urethrotome, *with stylet* complete........... | 10 | francs. |
| Large " (opening like scissors)....... | 30 | " |
| Bougies (to fit the above) according to size, from 1¼ to ½ | | " |

In connections with the foregoing subject is the method employed by the same surgeon to measure the distances between strictures where there are several. This is by introducing bougies of a small calibre completely through the strictures, each bougie terminating in a heart or olive-pointed head. Owing to the shape of this head it is readily *introduced,* bnt upon its withdrawal, at every stricture it "cathes," thus giving an indication to the manipulator, who ties a string upon the free portion of the instrument whenever an obstruction is felt, the distances between the strings denoting the separation of the strictures. Of course, due regard must be had to the comparative extension of the penis at the time, and some fixed point for comparison be settled upon before hand.

*The "electric cautery."*—Among the novelties of the day, is the improved "electric cautery," by means of the "pile of Middledrof" containing six "elements of Bunsen." This beautiful and neat means of applying the actual cautery has been introduced in Paris within the past two months. The principles involved are not new, the construction of the present apparatus unites the advantages of simplicity, portability and ease of administration, combined with the perfect control that the operator has over the agent. The whole is contained in a box, say 22 inches ⋈ 10 ⋈ 12. It is furnished by Charriére, complete with all the instrument for cauterization, section of tumors, etc., for 350 francs (say $70.) Charriére informs me that he expects to be able shortly to furnish the apparatus much cheaper, and also inclosed in smaller boxes.

The advantages of the "electric cautery" are double. In the first place, in general practice, the bad effect upon the minds of the patient and friends, to a great extent controls the application of the actual cautery as heretofore used, and in a manner hampers the will and desires of the medical practitioner. By using the *electric* apparatus the desired result can be obtained without shocking the non-professionel observer by the horrible sight of "red-hot pokers," and such things; and the patient may frequently consent to be "burned by electricity," when the mere mention of the "fer rouge" would almost secure the dismissal of the enterprising practitioner recommneding it.

The second good effect, in fact a *real* advantage, is that by using the present contrivance, the amount of heat can be exactly regulated, and a 'red" or a "white" heat generated and kept up at *will.*

Nélaton has recently used the "electric cautery" several times, at his cliniques, and expresses, himself very much pleased with it.

The ordinary actual cautery is much employed by Maisonneuve, and as equally eschewed by Nonat, in the treatment of ulcers of the os uteri and cervix. I saw the former apply the cautery in case of schirrous disease of the same parts, but for what useful purpose, I am unable to imagine. Jobert, at the Hôtel Dieu' has a mania for treating not only ulcers of the os uteri, but also many other complaints with this agent.

In those cases where the hot iron is applied directly to an ulcerated os uteri, the patients generally suffer but little pain from the burning of the *womb itself*, but they sometimes complain bitterly of the suffering induced by the *sides of the speculum* getting hot, and burning or smarting the *vagina.*

*The injection of iodine into the knee-joint, as a cure for chronic synovitis.* This plan of treatmeut is not unfrequently adopted; but the most that can be said in its favor is, that some of the patients operated upon have recovered. The only case in which I have seen the method used, was that of a young woman who had suffered from chronic synovitis of the right knee for a long time. On the 25th of January, after drawing off the superabundant synovial fluid, Chassaignac threw an iodine injection into the cavity of the joint thus emptied. The operator in his remarks at the time, said that as the only danger to be feared was from excessive inflammation, the after treatment would be confined to such means as would more effectually keep down and prevent undue inflammatory action. In addition to ordinary antiphlogistic measures, he relied greatly upon keeping the parts in a state of absolute quiet. Accordingly he confined the limb to a sort of extemporaneous inclined plane.

I heard from the patient at the expiration of a week, at which time she was in a very fair condition—everything apparently going on as favorably as could be wished. She stated that after the first two days she suffered but little pain. I will watch the result, and report it hereafter, should I continue my Paris correspondence with the Journal.

The French surgeons advocating this treatment say that in thus treating chronic synovitis of the knee-joint, except where untoward symptoms supervene, the result is simply an alteration of the secreting condition of the synovial membrane, *without the production of adhesions.* They also say that the parallel treatment of hydrocele by iodine injection, is in the majority of cases, *not* usually followed by adhesive inflammation, as is generally supposed, but simply by an altered condition of the secretive functions of the membrane.

Having thus given a rapid sketch of the principal novelties and "hobbies" at present in vogue in Paris surgical practice, I now leave the consideration of parallel matters in the practice of medicine proper, for a subsequent article. But before finishing the present paper, I feel constrained to make a few general observations on matters not properly embraced under its healing.

In the first place, a medical man, in viewing the systems and practice of foreign countries, necessarily compares what he there sees with what he has been in the habit of seeing at home: this has been my own case, and I hope that it will not be considered presumption if I recount a few of the things that most astonish me.

Foremost among these are the wretchedly ventilated wards of the principal Parisian hospitals, with the marked and only exceptions of the Lariboisière and Beaujon. The close; almost suffocating, wards of the Hôtel Dieu, Charité, La Pitie, and "Faculté" are a standing reproach to this nation, believing itself and boasting that it is at the head in medicine as well as in the other sciences. Even the Lariboisière itself is not, in my opinion, nearly as well ventilated or arranged as the New York Hospital, or several other kindred institutions in America. With the two exceptions mentioned, the hospitals of Paris are deficient in respect to ventilation, to an appalling extent. This readily accounts for the vast amount of erysipelas, phlebitis, etc.—the incubi upon Parisian surgery—which continually exist.

But there are other points which attract attention and which are at variance with most of our preconceived ideas. Wounds are enveloped in thick coverings of picked lint, compressed, etc., and appear to be thus kept *too warm*; at the same time but little care is taken to keep them

*clean* Cold water dressings are very rarely employed, and in some of the hospitals seem to be entirely eschewed. Evaporating lotions I have seen used but once! Several times I have noticed an eminent surgeon, while dressing an ulcerated surface, apply the sponge directly upon the *granulations!* yet he takes but little pains to wash or keep the skin *adjacent* to the discharging sores clean. This being generally covered with the dried, stinking decomposing secretions of the ulcers, is foul and disgusting.

The practice of keeping stinking wounds muffled in thick dressings, and under warm bed-clothes, does not strike me as consistent with good surgery and when combined with the bad ventilation of the wards themselves, already alluded to, must exert a very unfavorable influence upon the patients, and, to a certain extent, accounts for the frightful mortality which it is well known exists in the French hospital.

In hospital practice, simple cerate is the most freqent ointment employed. It is much the same as that used by us, except that the wax entering its composition, is the *unbleached*, yellow kind, I suppose, because it is cheaper—as the druggist shops generally supply the *white* cerate. It is also more fluid than with us which allows its use for lubricating bougies, and other instruments, and the hands for vaginal examinations. It is commonly emplyed for these purposes, and has the advantage over plain olive oil of not *dripping*, thus saving the bed-clothes, carpets, etc.

The treatment of fractured bones at present in Paris is worthy of the days of the enlightened "Frère Jacques," and a disgrace to the present French surgeons. I have scarcely seen a single case of fracture, especially of one of the long bones, that has not united with positive deformity, much of which might have been prevented by the proper application of *common sense.* There is scarcely a second-rate surgeon in the United States that would not blush to turn such "cures" from his hands, and at the delightful prospect of a civil suit for maltreatment. To enlarge upon this subject will occupy more room than can be spared; suffice it to say, that I am, in the above statment, echoing the views of every American student with whom I have conversed upon the subject.*

*The use of anæsthetics.*—Ether is not used at all, chloroform being solely relied upon. This latter article is almost invariably adminis-

* A friend walking through a ward of one of the large hospitals, saw a case of rather recent fracture of the thigh. The only treatment pursued was to order the man to lie flat on his back. A turn or so of a bandage was made around the foot, and then the ends pinned to the corner of the bed. This was the only substitute for entension, conter-extension and the other means for fulfilling the idea still in vogue in America, but apparently obsolete in France, of keeping fractured bones in apposition.

tered, even by Nélaton, who is considered by many to be the ablest and most complete surgeon in Paris. Fear of the evil consequences of chloroform causes the surgeons to be so cautious and timid in its administration, that the majority of the patients operated upon are but partially placed in a state of anæsthesia, and almost invariably during the course of an operation, the violent shrieks or struggles of the victim show that the consciousness of pain is scarcely diminished, while the intoxicating effect of the vapor destroys for the time his reasoning faculties, and frequently renders him very unmanageable. No indication is fulfilled by thus using the article, and it seems to me that rather than *half* use it, it had better be dispensed with entirely.

Chassaignac, who alone among the Parisian surgeons appears to have studied the modus operandi of chloroform, regulated its application with a great deal of nicety and discrimination—guiding himself by carefully watching the pulse and respiration. His patients rarely make a disturbance during the operation, and for the most part seem thoroughly unconscious of pain. After an operation in which the anæsthetic has been used, the last mentioned surgeon is very particular to have the patient carried back to the ward, with the *feet elevated* and the *head down.*—He fancies that the circulation in the brain is thus better kept up. The old man, despite his "hobbies," seems to be a very sensible, clear-minded surgeon, and an enthusiast in his profession. His lectures, interesting and practical, are delivered in such clear and measured accents, that his clinique is quite a favorite with English and American students.

Among the operations of more or less interest lately performed in Paris, besides those already mentioned, are the following:

*By Maisonneuve at La Pitié.*—Operation for fistula *in ano* with the view to complete cure. In this case the sides of the fistulous track were "scooped out" with the knife, the sphincter having been first divided.

Operation in December of "resection" [exsection] of the head of the femur. This was the case of a young woman suffering from morbus coxarius. The patient — two months having elapsed—is doing well, although much reduced by suppuration and long confinement. January 22d. Operation to produce an artificial anus at the bottom of the descending colon. This was the case of a patient suffering from complete malignant stricture of the rectum. The operation will certainly prolong the days of the wretched creature a little longer, and has given him great temporary relief, as the symptoms produced by the accumulated fæces were distressing and threatening.

The same surgeon has lately operated on a woman for femoral hernia. The strangulation was of eight days' standing, and, as a consequence, gangrene had completely taken place. The operation consisted simply in laying open the mass by a free incision. Maisonneuve's plan of treating strangulated irreducible herniæ is rather peculiar. If the strangulation has existed under forty hours he operates in the usual manner, and reduces the gut; but if it has lasted over that time, he does nothing until the seventh or eighth day, at which time he simply makes the incision as in the case given. An artificial anus, the result, he considers far preferable to the risk of general peritonitis should the gut be returned too early. He also waits, as we have seen, for a sufficient time to allow the firm adhesion of the gut at the point of departure, which otherwise would perhaps be drawn in by peristaltic action.

Maisonneuve is very partial to a method of removing malignant tumors in the submaxillary and carotid regions, by caustic. His method is to use "*flèches*" of the paste of canquoin (composed of chloride of zinc and wheat flour paste). These "*flèches*" are simply pegs the composition of which kiln-dried becomes quite hard; tapering to a sharp point at one end, their bases are from two to three lines square, and their length varying from one to one and a half inches. In action they are analogous to the common "trochisques" of corros. sublimate. When the "*pâté de canquoin*" is required simply for an external application, it is made of equal parts of the dough and the chloride, but in making the "*flèches*" two parts of flour paste are necessary to one of the salt; this is in order to secure the proper hardness and firmness when dry. After being dried they should be kept in a closely stoppered bottle.

To remove a tumor with the "*flèches*" they should be introduced into small lancet holes (through the integument), situated about half an inch apart around the circumference of the tumor. They should then be pushed obliquely backwards and inwards their entire length, their points converging towards a common centre. The caustic thus applied acts very effectually, but operating for a period of days subjects the patient to long continued suffering. If the whole of the tumor is not removed at first, fresh fleches are introduced in the same manner. Should the tumor be large, the cavity after its removal is appalling. Several of the cases that I have seen undergoing this treatment have been attacked by erysipelas, and one was fatal. The cause of death in this case was a hemorrhage of one of the branches of the external carotid occurring in a patient already much enfeebled by suppuration and long suffering.

The advantages which Maisonneuve thinks justify the removal of cancers by this method, are that they can be thus destroyed in places where the intricate relations with large blood vessels and nerves render attempts to "dig" them out with a knife hazardous, and unsatifactory.—As opposed to the foregoing, we find that the constitutional and *real* suffering during the action of the caustic is very great—severe hemorrhages *sometimes* take place, and erysipelas, facial paralysis, and other complications frequently occur. Should not these facts deter the conscientious surgeon from resorting to such means, especially when the chances of ultimate benefit to the patient are so remote, even though the tumor be successfully removed the first time?

At the Hôtel Dieu there have been but few operations during the winter. It has suffered much from erysipelas, typhoid fever and kindred disorders. The only operations of especial interest have been one or two attempts at curing vesico-vaginal fistula. These cases are a hobby with Jobert, who relies entirely upon the use of the interrupted suture. His other hobby is to use the actual cautery upon nearly everything. He has upon his "public days" two furnaces filled with red hot irons, which he uses with an unflinching hand for the benefit of suffering humanity, and the edification of the students following him.

At the Charité there has also been a great dearth of interesting surgical cases during the winter. Velpeau gives his ordinary tri-weekly clinical lectures as usual, to a very limited number of students.

Nélaton at the Hospital of the Facultly, is the great attraction to surgical students. With a happy faculty of explaining himself clearly, combined with a thorough understanding of his subject, he unites the common sense of a good practitioner and the cultivated manners of a gentleman.

His lecture-room is crowded daily, many students being obliged to stand throughout the lecture. During the past winter he has performed several interesting operations, removing tumors, etc. I saw him take out a diseased testicle very nearly. After the incision through the scrotum and tunica vaginalis with a scalpel, he finishes the operation with *scissors*. This is his ordinary plan, cutting the cord little by little, and tying each bleeding vessel as it is divrded. Among many practical remarks which continually enrich his lectures was one that struck me as well worth recording. He said that it is his rule never to attempt to put a *lateral* ligature upon the internal jugular, for the reason that nearly, if not quite, always the included portion at the end of four *or* five days *sloughs out leaving a larger hole than the first wound*, from which the sudden bursting out of a violent hemorrhage speedily proves fatal.

In the present article I have endeavored, in an impartial manner, to present the facts as they stand, for the most part leaving its readers to draw their own conclusion concerning the efficacy and value of the different methods spoken of that are now in vogue in Paris, compared with those that they are familiar with at home.—*N. Y. Jour, of Med.*

